# Remote sensing image super-resolution using multi-scale convolutional sparse coding network

**DOI:** 10.1371/journal.pone.0276648

**Published:** 2022-10-26

**Authors:** Ruihong Cheng, Huajun Wang, Ping Luo

**Affiliations:** College of Geophysics, Chengdu University of Technology, Chengdu, Sichuan, China; Tezpur University, INDIA

## Abstract

With the development of convolutional neural networks, impressive success has been achieved in remote sensing image super-resolution. However, the performance of super-resolution reconstruction is unsatisfactory due to the lack of details in remote sensing images when compared to natural images. Therefore, this paper presents a novel multiscale convolutional sparse coding network (MCSCN) to carry out the remote sensing images SR reconstruction with rich details. The MCSCN, which consists of a multiscale convolutional sparse coding module (MCSCM) with dictionary convolution units, can improve the extraction of high frequency features. We can obtain more plentiful feature information by combining multiple sizes of sparse features. Finally, a layer based on sub-pixel convolution that combines global and local features takes as the reconstruction block. The experimental results show that the MCSCN gains an advantage over several existing state-of-the-art methods in terms of peak signal-to-noise ratio and structural similarity.

## Introduction

Many remote sensing applications rely on high-resolution (HR) images with rich details, such as target detection and recognition [1–4], classification [5–9], and segmentation [9, 10]. However, some remote sensing satellites only provide images with low spatial resolution, which do not meet practical requirements in real-world scenes. Image super-resolution (SR) attempts to recover the HR image from the related low-resolution (LR) image. Therefore, SR is an essential topic in remote sensing. SR methods can be divided into multiple image super-resolution (MISR) and single image super-resolution (SISR). We pay more attention to the SISR because it is a well-known ill-posed inverse problem that the same LR images have multiple HR solutions [11].

The SISR problem can be solved by using three different methods: interpolation-based, reconstruction-based, and learning-based methods. Interpolation-based methods, such as bicubic interpolation (Bicubic), bilinear interpolation (BI), etc., are simple to implement. However, their performance is limited to a few smooth images, and their inability to recover high-frequency information limits their application [12]. The reconstruction-based methods perform image SR by employing a model of the degradation relationship between HR and LR images. However, they could only improve small magnifications because these images are severely lacking in high-frequency detail information [13, 14]. Yang et al. provide a comprehensive review of more SR methods [15]. Learning-based approaches are classified as sparse representation-based or deep-learning-based. Although sparse representation-based methods can recover high-frequency information by using prior knowledge, they are computationally complex and require massive computing resources [12, 16, 17]. Deep-learning-based methods directly learn an end-to-end mapping between low and high-resolution images, and significant improvements were observed [18].

Recently, convolutional neural networks (CNNs) have demonstrated remarkable performance in the SR problem [19, 20]. Dong et al. propose the SR convolutional neural network (SRCNN) model that applies CNNs to the SR problem for the first time [19]. Therefore, many studies pay attention to developing a more efficient network to learn the mapping between LR and HR images [21–25]. SRCNN was firstly introduced into remote sensing images SR by Liebel and Körner [26]. Li et al. introduce a local-global combined networks (LGCNet) super-resolution algorithm for remote sensing images [11]. It employs a “multifork” structure to learn multilevel representations of remote sensing images, including both local details and global environmental priors. Qin et al. introduce multiscale convolution neural network (MSCNN) to implement remote sensing SR [27]. Li et al. propose a Multi-scale residual network (MSRN) model for SR that takes advantage of multiscale image features [28]. Huan et al. propose a pyramidal multiscale residual network (PMSRN) model by use of multiscale dilation residual block and hierarchical feature fusion structure [12]. Li et al. propose a network combining inception residual attention network (IRAN) and channel attention, spatial attention to obtain multiscale features [29]. This method can comprehensively learn the features of remote sensing images, but it increases the complexity of the model. These networks are learned knowledge about SR from training data and ignore people’s domain expertise of images, such as natural image prior and image degradation model.These models outperformed in terms of peak signal-to-noise ratio (PSNR) and structural similarity (SSIM). Nonetheless, all of these models tend to build deeper and more complicated network structures, implying that training these models requires more resources, time, and tricks.

Inspired by the progresses of deep learning, some deep learning models that combined sparse coding (SC) are proposed [23, 30] and have a wide application in SR. According to the assumption of SC, HR images can be reconstructed with a sparse representation of the learned dictionaries. If the dictionaries are properly defined, the LR and HR image patches can be represented in terms of a pair of overcomplete dictionaries using the same sparse linear coefficients [23]. The dictionary pair can be learned alternatively with the inference of training patches’ sparse codes in their joint space [31] or through bilevel optimization [32]. The sparse coding coefficients are generally solved by the iterative shrinkage and thresholding algorithm (ISTA), but the results significantly depend on hyperparameters [22]. Because of the relationship between ISTA and neural network, literature [23] proposed a sparse coding based network (SCN) for image SR, which combines domain expertise and deep learning to design better deep model architectures. Thus, all of SC model parameters can be learned through training instead of needing analytical solutions. It is proved that sparse coding based super resolution can be treated as an end-to-end training of model components by convolutional neural network [23]. Based on the SCSR algorithm and VDSR network, an image super-resolution reconstruction algorithm is proposed in [30], which combined with multi-residual network and multi-feature SCSR (MRMFSCSR). It can improve the image detail information and maintain the geometric structure information at the same time, developing a better reconstruction image.

In summary, the current popular approaches typically have the following problems: 1) Difficulty in replication: Some SR reconstruction methods contain multiple network layers, which necessitate the use of complex hardware. Besides, the same model obtains varied performances by employing alternative training tricks, implying that the gain in performance may not be due to a change in model architecture, but to the application of some undiscovered training techniques. Because of these qualities, recurrence of these network models is difficult. 2) Inadequacy of features utilization: Most approaches frequently fail to make full advantage of the LR image attributes with only raising the depth of the network instead. It is critical for the network to understand how to make full use of these features to rebuild HR images. 3) Ignorance of domain expertise: The domain expertise can be used to design better deep model architectures, i.e. sparse coding model, etc. [23]. However, most networks are built with convolutional neural network, which means all their knowledge about SR are learned from training data. Therefore, in deep learning-based methods, people’s domain expertise of images, such as natural image prior and image degradation model, is largely ignored.

This article presents a novel multiscale convolutional sparse coding network (MCSCN) to solve the mentioned problems. It is shown that domain expertise can improve the SR performance [23]. Therefore, we adopt multiscale convolutional sparse coding module (MCSCM) for MCSCN, which combines the sparse coding and deep learning. Firstly, we use the MCSCM to obtain the different scales image features, which are referred to local multiscale features. Secondly, the outputs of each MCSCM are concatenated for global feature fusion. Finally, the combination of local multiscale features and global features can maximize the use of the LR image features. Contributions of this paper are as follows:

- Proposing a novel MCSCM. This module extracts multiscale features with stacking dictionary convolutional units, implements multiscale sparse coding using different convolutional kernel sizes, and adaptively improves image features extraction.
- Combining the convolutional sparse coding with deep learning for image SR. Based on dictionary convolutional units, we can conduct a feed-forward neural network to carry out the convolutional sparse coding. It can improve performance by consolidating the merits of convolutional sparse coding with the domain knowledge of deep neural networks.
- Conducting an objective evaluation on several representative and state-of-the-art SR methods with remote sensing image datasets.

## Materials and methods

**Fig 1:**
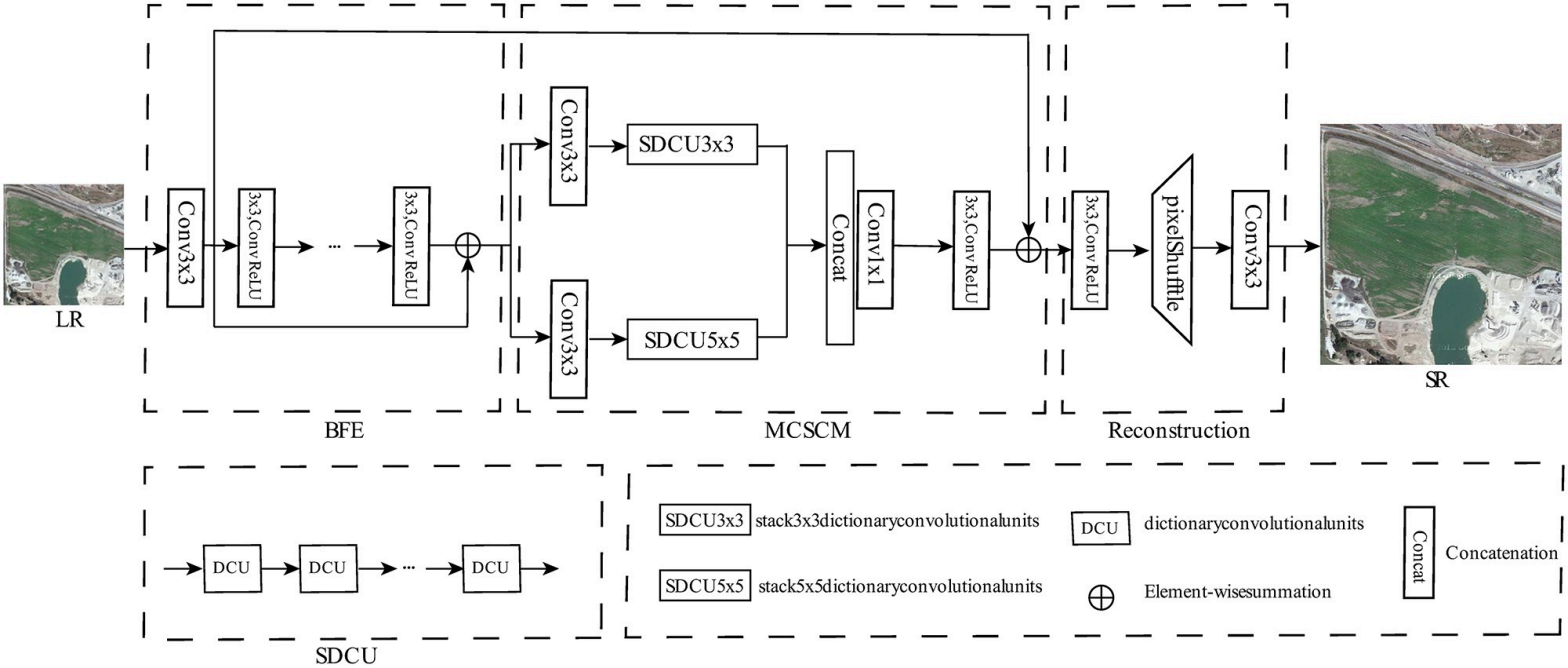
Architecture of the proposed MCSCN model. Our network consists of three main parts: BFE, MCSCM, and reconstruction module. We introduce a MCSCM to complement the information and make full use of the different scales of feature information.

In this section, we will give a brief overview of the proposed networks and then present the details of each part. Fig 1 shows the architecture of the network. We apply a new network that combines conventional sparse features and deep learning to the image SR. Unlike most patch-based SR algorithms, our proposed network explicitly accepts LR images as input. Our model can be divided into three parts: the basic feature extraction (BFE), the multiscale convolutional sparse coding module (MCSCM) and the reconstruction module. Each of the modules is described in the following. Given the fact that sparse coding can be effectively implemented with generalized dictionary convolutional units (DCUs), it is straightforward to build a multi-layer neural network that extracts the sparse features. So we will firstly describe the DCUs.

### Dictionary convolutional units (DCUs)

Given an image $X\in R^{c\times h\times w}$ (c = 1 for gray images and c = 3 for RGB images) and q convolutional filters $D\in R^{q\times c\times s\times s}$, Convolutional sparse coding model (CSC) can be formulated as the following problem,

$$\begin{array}{c} \underset{z}{\text{min}}\frac{1}{2}\|X-D*x{\|}_{2}^{2}+\lambda g\left(z\right), \end{array}$$ (1)

where λ is a hyperparameter, * denotes the convolution operator, z is sparse feature maps, and g(·) is a sparse regularizer. This problem can be solved by iterative methods, and it is easily written as

$$\begin{array}{c} z^{k+1}⇐prox_{\lambda /\rho }\left(z^{k}+\frac{1}{\rho }D^{T}*\left(x-D*z^{k}\right)\right), \end{array}$$ (2)

where *ρ* is the step size and **D**^**T**^ is the flipped version of **D** along horizontal and vertical directions. Note that prox(·) is the proximal operator. If g(·) is the ℓ_1_-norm, the proximal operator is also soft shrinkage thresholding function. By the principle of algorithm unrolling, we can employ convolutional units to replace the filters and extend the proximal operator to activation function, [22] the Eq (2) can be rewritten as

$$\begin{array}{c} z^{k+1}=f\left(\text{BN}\left(z^{k}+conv_{1}\left(X-conv_{0}\left(z^{k}\right)\right)\right)\right), \end{array}$$ (3)

where we also take batch normalization (BN) into account.

**Fig 2:**
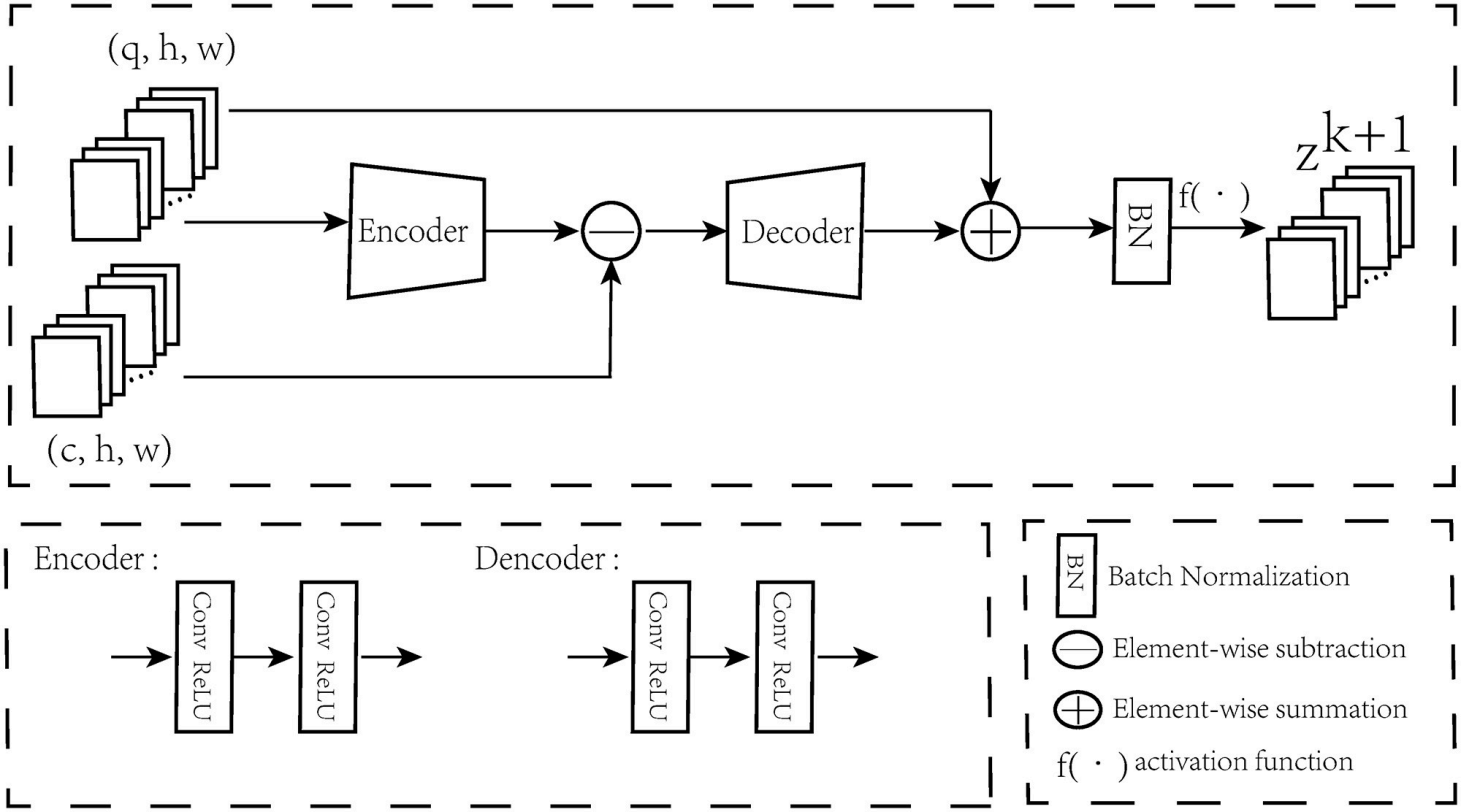
Architecture of DCU. It mainly contains encoder and decoder layers, and we can use different kernel sizes to achieve different scales of information.

The Eq (3) is called a dictionary convolutional unit (DCU). The implementation of DCU is shown in Fig 2. For the encoder module, we use convolution layers to maps the feature space into image space. And for the decoder module, we also use convolution layers to map the residual between the images and the reconstructed images from image space to feature space. By stacking DCUs, the original CSC model can be represented as a deep neural network. This process for CSC model can be regard as an iterative auto-encoder [22].

### Basic feature extraction (BFE)

BFE first embeds the LR image into the feature space and then lets the embedding feature pass through M mapping layers to obtain the output feature. We name the output feature from BFE as the base feature because we need to reconstruct the SR details by passing the feature through the MCSCM.

Given the input LR image, *I*^*LR*^ ∈ *R*^*h*×*w*×*c*^ where h and w denote the height and width of the image, respectively. We first define the embedding feature and M mapping layers as

$$\begin{array}{c} F_{e}=Conv_{3\times 3}^{3,n}\left(I^{LR}\right) \end{array}$$ (4)

$$\begin{array}{c} F_{i}=f_{3\times 3,ReLU}^{i}\left(F_{i-1}\right),i=1,2,\cdots ,M, \end{array}$$ (5)

where $Conv_{3\times 3}^{3,n}$ denotes a 3×3 convolution operation and the number of input and output channels are 3 and n, respectively. *F*_*e*_ is the embedding feature, $f_{3\times 3,ReLU}^{i}$ represents the *i*th mapping layer in BFE, and *F*_*i*_, *F*_*i*−1_ are input and output feature of the *i*th mapping layer.

Besides, we use local residual learning to integrate the features in BFE, so the entire BFE process can be formulated as

$$\begin{array}{c} F_{M}=f_{3\times 3,ReLU}^{M}\left(f_{3\times 3,ReLU}^{M-1}\left(\cdots f_{3\times 3,ReLU}^{1}\left(Conv_{3\times 3}^{3,n}\left(I^{LR}\right)\right)\right)\right) \end{array}$$ (6)

$$\begin{array}{c} F_{B}=f_{LRL}\left(F_{M},F_{e}\right), \end{array}$$ (7)

where *f*_*LRL*_(⋅) denotes the local residual learning operation and *F*_*B*_ indicates the output feature of the BFE module.

### Multiscale convolutional sparse coding module (MCSCM)

As we know, the performance of traditional ISTA algorithm for CSC model highly depended on the configuration of hyperparameters. The multiscale nature of the image is similar to that of human eyes observing an object. In order to detect the image sparse features at different scales, we propose multiscale convolutional sparse coding module (MCSCM). This module consists of numbers of DCUs with different scales in Fig 2. The Basic image feature **F**_*B*_ pass through the stacking dictionary convolutional units (SDCU) with different convolutional filters (with kernel sizes 3 × 3 and 5 × 5), respectively. The structure outputs **P**_1_ and **P**_2_ can be expressed as

$$\begin{array}{c} P_{i}=\text{SDCU}(F_{B};\;\Theta_{i})\;i=1,2, \end{array}$$ (8)

where SDCU(·) denotes the sparse coding for original feature **M**_0_ predicted using the CSC model with parameter set Θ_*i*_ (i = 1,2), respectively.

Additionally, the output of each SDCU contains distinct sparse features. These sparse features contain more information, and the computational complexity will be increased if using them directly for reconstruction. In order to adaptively make use of these hierarchical features, the bottleneck layer and 3 × 3 convolution is introduced by Xu et al. [22] and Li et al. [28]. The output can be formulated as

$$\begin{array}{c} F_{\text{LR}}=\omega_{3\times 3}*\left(\omega_{1\times 1}*concat\left(P_{1},\;P_{2},\right)+b_{0}\right)+b_{1}, \end{array}$$ (9)

where **P**_*i*_ (i = 1,2) represents the output of the *i*_*th*_ stacking DCUs, *w*_1×1_,*w*_3×3_ and *b*_0_, *b*_1_ represent 1 × 1, 3 × 3 convolution kernels and their biases respectively; Note that concat(·) is the concatenation operator.

### Image reconstruction

The LR inputs of the previous super-resolution methods are often upsampled to the same dimensions as HR using Bicubic. This approach will increase the computational complexity. The sub-pixel convolutional operation is widely applied to solve this problem in signal image super resolution [28, 33]. Furthermore, it is critical to discover a mechanism to combine the shallow and sparse features. As a result, a structure is constructed using Basic feature **F**_*B*_ and sparse feature of multiscale convolutional sparse model. As shown in Fig 1, the Basic feature **F**_*B*_ and sparse features from MCSCM respectively perform sub-pixel convolutional layer and rearrange the image tensor with dimensions H × W × C*r*^2^ as *r*H × *r*W × C. Then, the features are reconstructed as SR image after 3 × 3 standard convolution. It is proved that the reconstruction structure makes use of the original feature information and prevents information loss [22].

## Results and discussion

In this section, we evaluate the performance of our model on several benchmark test datasets. Firstly, we explain the dataset used in the training and testing process, and then give implementation details. Secondly, we compare our model with several state-of-the-art methods. Finally, we introduce the result of our model and give some result analysis.

### Datasets

We choose two datasets with plentiful scenes to verify the robustness of our proposed method, namely aerial image dataset (AID), UCMerced Land Use (UCM).

The AID is a large aerial image dataset that collects sample image from Google Earth images. It contains more than 10,000 images of 30 land-use scenes, including river, mountain, farmland, pond, and so on. All the images of each category were carefully selected from different countries and regions of the world. Therefore, the diversity in the class of the data has been strongly increased. We randomly choose 20% of the total number as the testing set, and the remaining 80% as the training set.

The UCM dataset was released by the University of California in 2010. It contains 21 types of remote sensing scenes such as medium residential, airplanes, storagetanks, and parking lots and so on. Each class includes 100 pictures. We also randomly selected 80% of the images as the training set and 20% as the testing set.

During testing, we also choose the RSSCN7 dataset and the test dataset with 20 images (called Test20 for short) used by Fernandez-Beltran et al. as testing set [34].

### Implementation details

During training, the image data is augmented by random rotation, and flips to expand the dataset. We generate the LR images by the Bicubic and extract the LR patches with the size of 48 × 48. We set the training epochs as 1000. We train our model with the ADAM optimizer by setting the learning rate to 0.0001, *β*_1_ = 0.9 and *β*_2_ = 0.999. In our model, we use 4 DCUs for SDCU and the output of MCSCM has 128 features. Our model directly trained and tested in RGB color space. In addition, the upscaling factors: ×2, × 3 and ×4 are used for both training and testing. We implement MCSCN with the PyTorch framework and train them using the NVIDIA RTX 2080ti GPUs.

### Evaluation metrics

The evaluation metrics for experiments results contain peak signal-to-noise ratio (PSNR), structure similarity (SSIM) and spectral angle mapper (SAM). Given a reference images I and a reconstructed image $\hat{I}$. The widely used metric is PSNR, defined as follows:

$$\begin{array}{c} RMSE\left(I,\hat{I}\right)=\sqrt{\frac{1}{K\cdot N}\sum_{j}^{K}\sum_{i}^{N}{\left(I_{i}^{j}-{\hat{I}}_{i}^{j}\right)}^{2}} \end{array}$$ (10)

$$\begin{array}{c} PSNR\left(I,\hat{I}\right)=20log_{10}\frac{255}{RMSE}, \end{array}$$ (11)

where the index j is used to identify each one of the K image bands and N is the total numbers of pixels in each image.

The SSIM is calculated as

$$\begin{array}{c} SSIM\left(I,\hat{I}\right)=\frac{\left(2u_{I}u_{\hat{I}}+c_{1}\right)\left(2\sigma_{I\hat{I}}+c_{2}\right)}{\left(u_{I}^{2}+u_{\hat{I}}^{2}+c_{1}\right)\left(\sigma_{I}^{2}+\sigma_{\hat{I}}^{2}+c_{2}\right)}, \end{array}$$ (12)

where *u*_*I*_ and $u_{\hat{I}}$ are the mean of I and $\hat{I}$, respectively, $\sigma_{I}^{2}$ and $sigma_{\hat{I}}^{2}$ are the variance of I and $\hat{I}$, respectively and $\sigma_{I\hat{I}}$ is the covariance of I and $\hat{I}$. *c*_1_ = (*k*_1_ *L*)^2^ and *c*_2_ = (*k*_2_ *L*)^2^ are the constants used to maintain stability. L is the dynamic range of the pixel value and *k*_1_ = 0.001 and *k*_2_ = 0.003. A higher PSNR and SSIM value represents a better image quality.

SAM considers each spectral band as a coordinate axis, and then it computes the average angle between the pixels I and $\hat{I}$. Its expression defined as

$$\begin{array}{c} SAM\left(I,\hat{I}\right)=\frac{1}{N}\sum_{i}^{N}arccos\frac{I_{i}\cdot {\hat{I}}_{i}}{\|I_{i}\left\|\right\|{\hat{I}}_{i}\|}, \end{array}$$ (13)

note that the ideal value of SAM is 0.

### Loss function

We choose the L1 loss (i.e. mean absolute error) as the loss function, since L2 loss (i.e. mean square error) penalizes larger errors, but it is more tolerant to small errors, and thus often results in too smooth results. The L1 loss can be formulated as

$$\begin{array}{c} L_{1}\left(\hat{I},I\right)=\frac{1}{hwc}\sum_{ijk}\left|{\hat{I}}_{i,j,k}-I_{i,j,k}\right|, \end{array}$$ (14)

where h, w and c are the height, width and number of channels of the evaluated images, respectively.

### Ablation experiments

We designed a set of ablation experiments to verify the effectiveness of the MCSCM structure, including the kernel sizes of the MCSCM and the number of stacked DCU units.

The ablation experiments about the kernel sizes of the MCSCM module are performed on x4 AID dataset, as shown in Table 1. We test single-scale and multiscale convolution kernel sizes for MCSCN to explain the impact of multiscale on reconstruction results. The effect of reconstruction may be improved to 0.57-0.76 dB by employing different scales of convolution kernels. A small-scale convolution kernel may extract local details, whereas a large-scale convolution can extract broader global features [12, 28]. We can gain more plentiful details by integrating features collected from different convolution kernels. Better results can be obtained by combining global and local multiscale features.

**Table 1:** PSNR comparison of different kernel sizes for the MCSCM.

Scale	Kernel sizes	PSNR(dB)
Single-scale	3 × 3	29.25
	5 × 5	29.06
Multiscale	3 × 3 and 5 × 5	29.82

**Fig 3:**
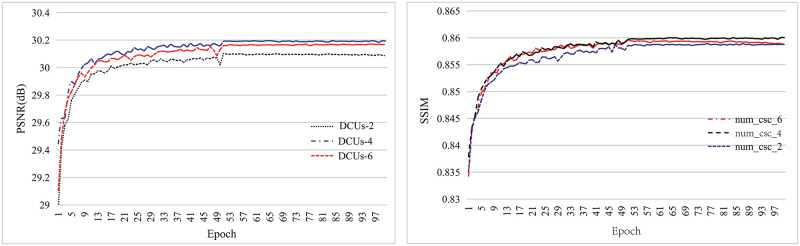
The PSNR and SSIM of the different number of stacked DCUs.

The ablation experiment about the number of stacked DCUs is shown in Fig 3. It is shown that the PSNR and SSIM results of 4 stacked DCUs are higher than that of 2 or 6 stacked DCUs, indicating that the use of 4 stacked DCU units has an effective performance to the proposed structure.

### Comparison with the state-of-the-art method

In this subsection, we compare our model with the FSRCNN [35], VDSR [36], LGCNet [11], EDSR [37] and IRAN [29] on the RSSCN7, UCM and Test20 datasets. The LGCNet and IRAN are representative SR models for remote sensing images, while the other methods are excellent models for natural scenes. All these methods are trained and tested under the same conditions for the sake of fairness.

**Fig 4:**
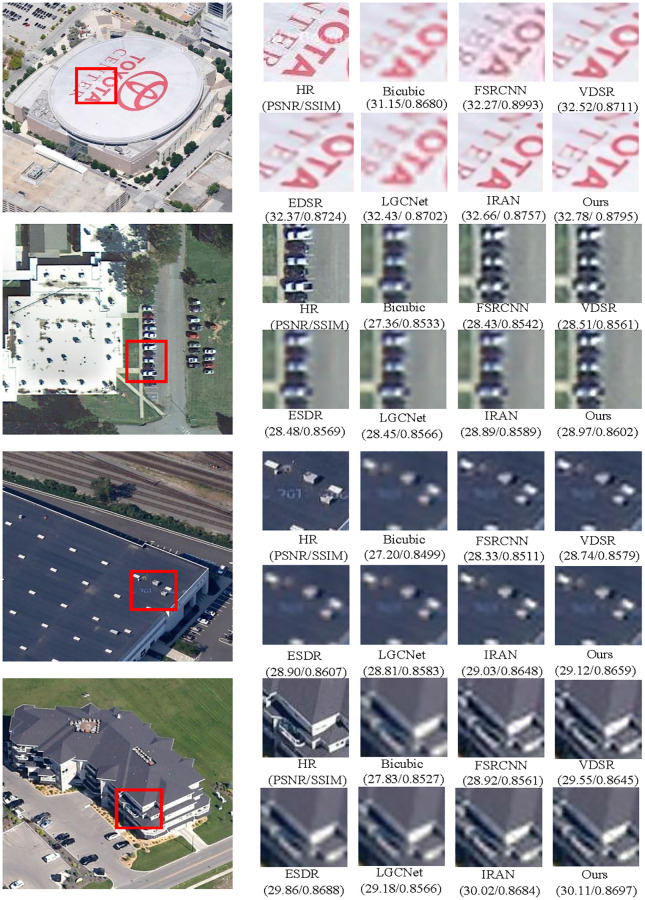
The visual comparison results magnified by an upscaling factor 4.

Table 2 shows the peak-signal-to-noise ratio (PSNR) and the structural similarity (SSIM) with the up-scaling factors ×2 and ×4 for the methods mentioned above on the RSSCN7 dataset, including Grass, Field, Industry, RiverLake, Forest, Resident, and Parking. The results in bold indicate the best performance methods. We have average PSNR gains of 0.126 dB and 0.121 dB for the up-scaling factors ×2 and ×4, respectively. Fig 4 shows the visual effect obtained by using our method and the compared methods on the RSSCN7 with up-scaling factor ×4. To improve contrast, a tiny region marked by the red rectangle is enlarged, and the enlarged image is shown on the right of the images. As observed in the local enlarged image, our approach produces images with more refined boundaries and richer textures than others. Obviously, it can be seen that our method is superior to the other compared methods.

**Table 2:** PSNR and SSIM comparison results among different methods. Boldface indicates the best performance and italics indicate the second-best performance.

Dataset	Scale	Metric	Bicubic	FSRCNN	VDSR	LGCNet	EDSR	IRAN	Ours
Grass	×2	PSNR	34.691	36.599	36.867	36.753	36.156	*3*6.516	**36.887**
Grass	×2	SSIM	0.9042	0.9315	0.9342	0.9331	0.9392	*0*.9440	**0.9551**
Grass	×4	PSNR	30.882	31.537	*3*1.844	31.713	31.065	31.102	**31.982**
Grass	×4	SSIM	0.7701	0.7932	0.7959	0.7952	*0*.8077	0.8051	**0.8953**
Field	×2	PSNR	33.522	34.778	35.01	34.854	35.368	*3*5.607	**35.711**
Field	×2	SSIM	0.8269	0.8541	0.8611	0.8567	0.8689	*0*.8739	**0.8916**
Field	×4	PSNR	30.367	31.051	31.352	31.186	31.591	**31.608**	*3*1.365
Field	×4	SSIM	0.7015	0.7184	*0*.7249	0.7215	0.7314	0.7318	**0.8488**
Industry	×2	PSNR	26.564	28.694	29.426	29.067	29.542	*2*9.715	**29.828**
Industry	×2	SSIM	0.8442	0.8926	0.9081	0.9201	0.9122	*0*.9168	**0.9179**
Industry	×4	PSNR	22.311	23.497	24.012	23.595	24.261	**24.294**	*2*4.328
Industry	×4	SSIM	0.6163	0.6819	0.7066	0.6918	0.7213	0.7219	***0*** **.7233**
RiverLake	×2	PSNR	32.112	33.57	33.843	33.827	34.128	*3*4.332	**34.417**
RiverLake	×2	SSIM	0.8901	0.9245	0.9215	0.9261	**0.9327**	0.9326	*0*.9311
RiverLake	×4	PSNR	28.356	29.235	29.504	29.326	*2*9.608	29.557	**29.634**
RiverLake	×4	SSIM	0.7708	0.7963	0.8016	0.7983	0.8066	*0*.8072	**0.8179**
Forest	×2	PSNR	30.191	31.535	31.567	31.601	31.691	*3*1.968	**32.018**
Forest	×2	SSIM	0.8521	0.8765	0.8719	0.8745	0.8823	*0*.8869	**0.8902**
Forest	×4	PSNR	26.256	26.868	26.934	26.879	*2*7.025	27.021	**27.033**
Forest	×4	SSIM	0.5706	0.6132	0.6189	0.6154	*0*.6249	0.6247	**0.6277**
Resident	×2	PSNR	25.562	27.612	28.241	27.936	28.387	*2*8.539	**28.616**
Resident	×2	SSIM	0.8357	0.8901	0.9012	0.8916	0.9043	*0*.9084	**0.9107**
Resident	×4	PSNR	22.019	22.365	22.816	22.511	23.045	*2*3.078	**23.126**
Resident	×4	SSIM	0.5868	0.6624	0.6872	0.6718	0.7012	*0*.7034	**0.7055**
Parking	×2	PSNR	26.079	27.673	28.465	28.129	28.706	*2*8.852	**28.931**
Parking	×2	SSIM	0.8193	0.8726	0.8879	0.8814	0.8954	*0*.9011	**0.9034**
Parking	×4	PSNR	22.453	23.189	23.647	23.402	**23.849**	23.778	*2*3.821
Parking	×4	SSIM	0.5952	0.6514	0.6754	0.6601	0.6904	*0*.6919	**0.6925**
Average	×2	PSNR	29.817	31.494	31.917	31.738	31.997	*3*2.218	**32.344**
Average	×2	SSIM	0.8532	0.8917	0.8980	0.8976	0.905	*0*.9091	**0.9143**
Average	×4	PSNR	26.092	26.820	27.158	26.945	27.206	*2*7.207	**27.327**
Average	×4	SSIM	0.6588	0.7024	0.7158	0.7077	0.726	*0.7266*	**0.7587**

**Table 3:** PSNR/SSIM comparison on remote sensing test datasets among different methods with up-scaling factor ×4. Boldface indicates the best performance and italics indicate the second-best performance.

Image	Metric	Bicubic	CSCN	FSRCNN	LGCNet	IRAN	ESRGAN	MRMFSCSR	MCSCN(ours)
Airplane25	PSNR	26.62	28.40	27.86	28.55	**29.93**	28.68	29.81	*29.90*
	SSIM	0.8903	0.9144	0.9122	0.9285	*0.9357*	0.9182	0.9329	**0.9449**
	SAM	0.598	0.560	0.541	0.540	0.385	0.391	*0.370*	**0.368**
Airplane85	PSNR	27.10	28.52	27.97	28.67	29.61	29.13	*29.75*	**29.78**
	SSIM	0.8833	0.9084	0.8998	0.9175	0.9300	0.9211	*0.9328*	**0.9391**
	SAM	0.611	0.608	0.528	0.477	*0.360*	0.421	0.406	**0.352**
Overpass02	PSNR	25.52	27.01	26.66	26.89	**28.87**	28.39	*28.86*	28.85
	SSIM	0.8271	0.8815	0.8583	0.8632	0.8832	*0.8865*	0.8836	**0.8921**
	SAM	0.675	0.538	0.427	0.358	**0.360**	0.362	0.366	*0.361*
Overpass12	PSNR	27.32	28.53	28.67	29.18	*29.72*	29.22	29.70	**29.78**
	SSIM	0.8551	0.9037	0.8824	0.8953	*0.9068*	0.8920	0.9066	**0.9115**
	SAM	0.627	0. 594	0.493	0.362	0.357	0.361	*0.345*	**0.338**
Test20 dataset	PSNR	26.90	28.82	28.31	28.97	*29.82*	29.19	29.80	**29.85**
	SSIM	0.8632	0.9035	0.8905	0.9034	**0.9144**	0.9024	0.9126	*0.9127*
	SAM	0.641	0.532	0.480	0.364	0.324	*0.320*	0.326	**0.317**

**Fig 5:**
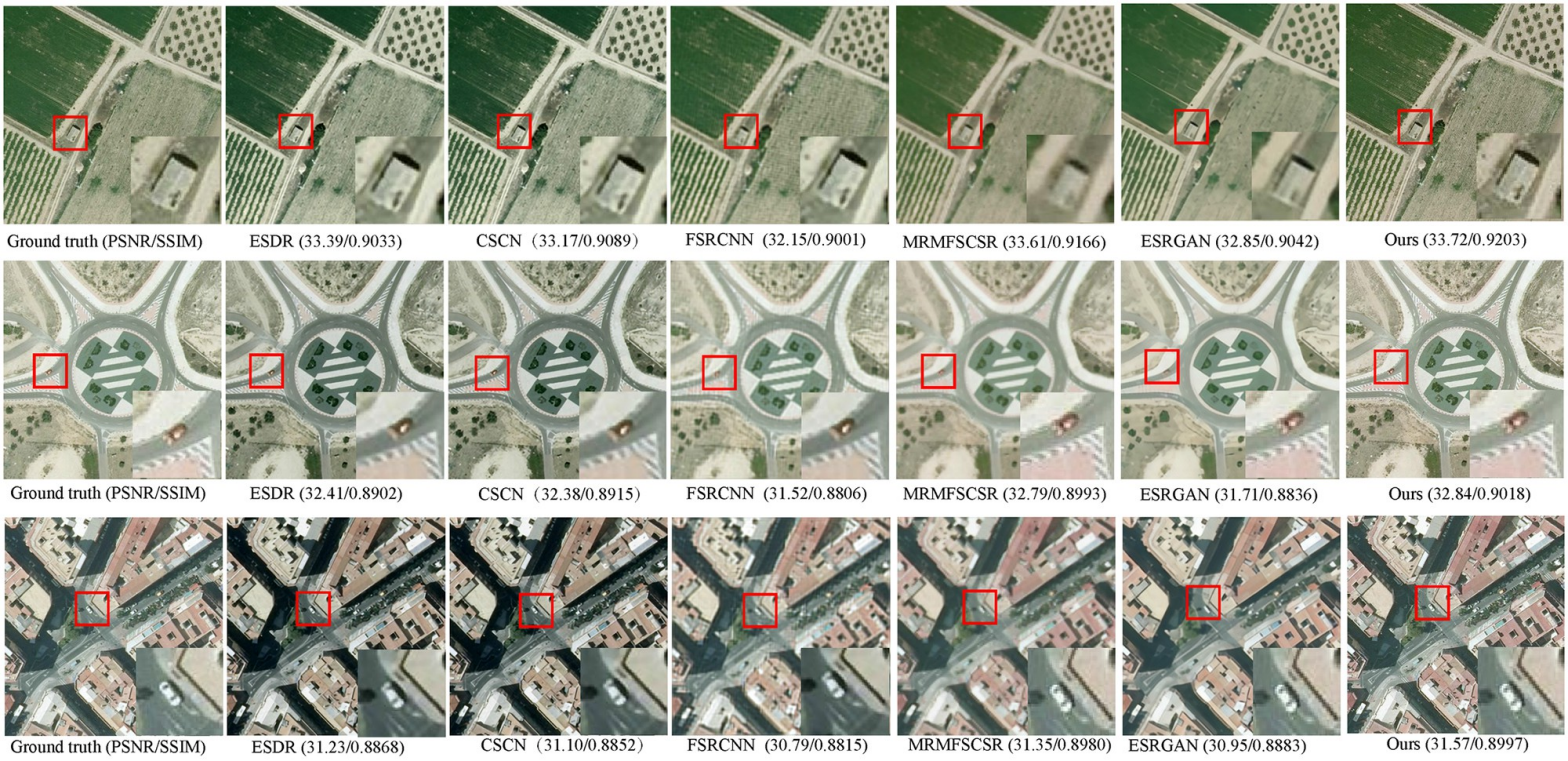
The comparative results of Test20 dataset magnified by an up-scaling factor 3.

**Fig 6:**
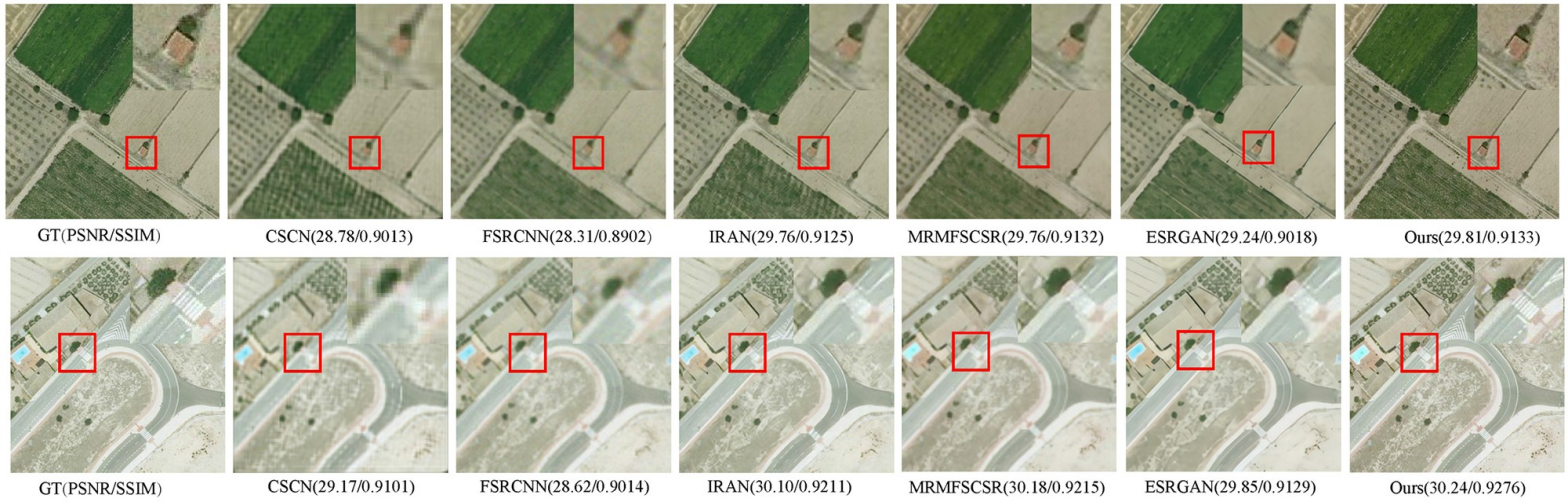
The visual comparison of Test20 dataset SR obtained using different methods with an up-scaling factor 4.

Furthermore, we also compare our model on the UCM test images and Test20 dataset with several methods stated before and additional MRMFSCSR [30] and ESRGAN [38]. Table 3 provides the values of PSNR, SSIM and SAM on the 4 test images from UCM dataset and the all images from Test20 dataset with up-scaling factor ×4. As a whole, it can be seen that the PSNR and SSIM of our model outperform the compared approaches. Figs 5 and 6 show the visual comparison of the previous methods in the Test20 with up-scaling ×3 and ×4, respectively. It is observed that our model produces finer details, and the detailed information of the reconstructed SR image is more closely match the ground truth images. It demonstrates that our model achieves competitive performance compared to other methods.

### Comparison on model size

**Fig 7:**
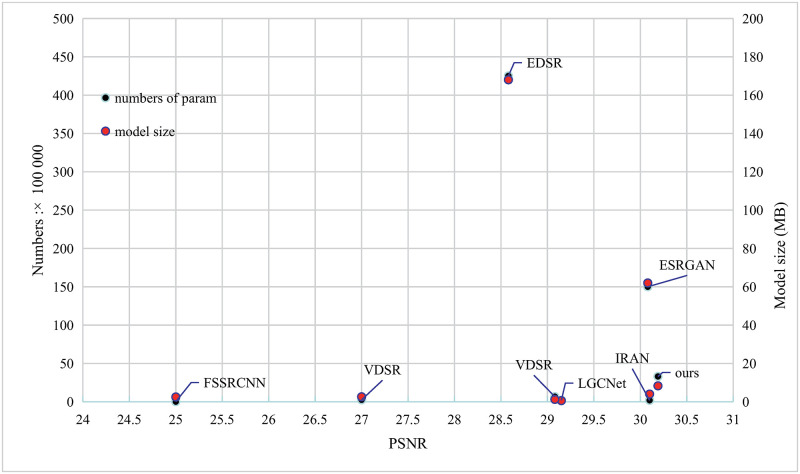
Comparison between model complexity and image quality. The left vertical axis is the number of parameters, and the right vertical axis is the size of the model file.

We choose some of the state-of-the-art SR approaches for the computation complexity comparison, including FSRCNN, VDSR, LGCNet, IRAN, EDSR and ESRGAN. Note that we use the models and network setting that the authors claimed the best in their experiments. Fig 7 shows the comparison of parameters and PSNR for 4× SR for AID dataset. The right bottom corner represents good with better PSNR and less model complexity. As one can notice, our method can achieve higher PSNR than EDSR and ESRGAN with much fewer number of parameters.

## Discussion

### The limitations of this research

The LR images of the train data are degraded for using bicubic interpolation. Actual LR images have a different distribution compared to the ones generated synthetically using bicubic interpolation. As a result, our methods can’t be used for blind SR. there are very few works whose target SR rates are higher than 8× [12]. In such extreme upsampling conditions, it becomes challenging to preserve accurate local details in the image. Therefore, this situation also exists in our model. The sub-pixel layer may result in some artifacts near the boundaries of different blocks. On the other hand, it may cause unsmooth outputs [39]. The research of deep learning in the field of remote sensing image SR can be carried out in the following aspects in the future:

- There is still a scarcity of specific data sets for remote sensing SR. Future research can be done to try to create a remote sensing SR dataset with abundant LR and HR images. Besides, we can also use blind SR methods for remote sensing images.
- Recently, most upsampling methods are the bicubic interpolation. To overcome the shortcoming of this, we can learn upsampling in an end-to-end manner [39]. We will use these learning-based layers as upsampling methods for our method in the future.
- SR performance can be improved by combining multi-stage and multiscale features. As a result, it points in the direction of increased SR rates. In the future, we can observe deeper into these scenarios.

## Conclusion

In this paper, we put forward a novel SR model for remote sensing images, which combines the convolutional sparse coding and deep network. We employ the multiscale sparse coding module to obtain multiscale sparse features, which we then fuse with global features to derive abundance features. By using sparse coding knowledge, we can gain considerable improvement over the several deep learning models.

In the future, we plan to apply the MCSCN approach to additional issues where spare convolutional coding might be beneficial. The interplay of deep networks for low- and high-level vision tests will also be investigated. We will also research this model employed in multi-spectral images.
